# Primary hydatid cyst of the pancreas of the child: a case report

**DOI:** 10.11604/pamj.2017.27.229.12853

**Published:** 2017-07-28

**Authors:** Achraf El Bakkaly, Nour Merouane, Omar Dalero, Houda Oubeja, Mounir Erraji, Fouad Ettayebi, Hicham Zerhouni

**Affiliations:** 1 Pediatric Surgical Emergency Department, Children’s Hospital, University Hospital of Ibn Sina, Faculty of Medicine Mohammed V, BP 6527, Street of Lamfadel Cherkaoui Rabat Institut, Rabat, Morocco; 2 Department of Pediatric Surgery, University Hospital of Mohammed VI, Oujda, Morocco

**Keywords:** Pancreas, hydatid cyst, child, treatment

## Abstract

Primary pancreatic hydatid lesions are very rare with an incidence of less than 1% in the adult population. We report an observation of a 5-year-old girl who consulted for isolated abdominal pain occurring for 2 weeks without vomiting, transit disorders or jaundice and evolving in a context of conservation of the general condition and apyrexia. Clinical examination and preoperative imaging have suggested the diagnosis of a choledochal cyst or duodenal duplication rather than a hydatid cyst of the pancreas due to the presence of a cystic hepatic image projecting into the liver hilum. During the procedure, a hydatid cyst was found occupying the head of the pancreas. Primary hydatidosis of the pancreas in children is extremely rare. Possible sources of infection include: blood diffusion, local spread via biliopancreatic ducts and peripancreatic lymphatic invasion. In the endemic areas, hydatid disease should be mentioned in the list of differential diagnoses of cystic lesions located around the biliopancreatic junction in children.

## Introduction

Pancreatic localization of the hydatid cyst is rare, even in countries where hydatid disease is endemic. The hydatid cyst of the pancreas accounts for less than 1% of all the sites, which may explain the difficulties of diagnosis. This location affects the child exceptionally [1]. We report an observation of this localization occurring in a 5-year-old girl and revealed by abdominal pain. Through this observation and a review of the literature, we discuss the diagnostic difficulties and the modalities of the surgical treatment of this unusual localization of the hydatid disease.

## Patient and observation

**Figure 1:**
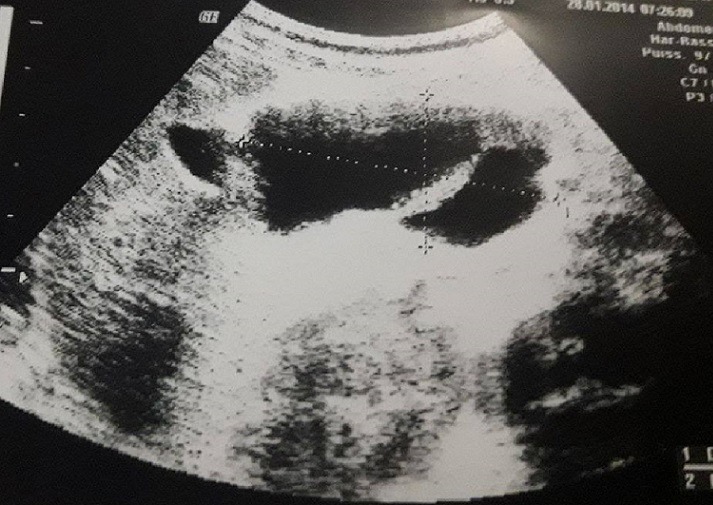
Ultrasound image showing a hepatic hilar multilocular cystic mass in contact with the anterior surface of the head of the pancreas

**Figure 2:**
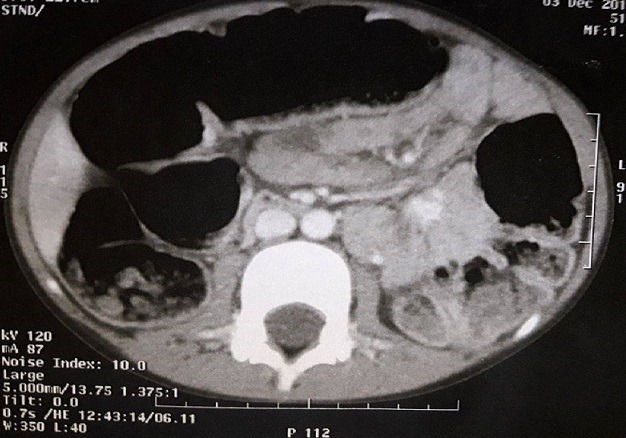
Abdominal angioscan: lesional compartmentalized cystic process in contact with the second duodenum, with a thickened wall making 57mm/31mm pushing back the pancreas

**Figure 3:**
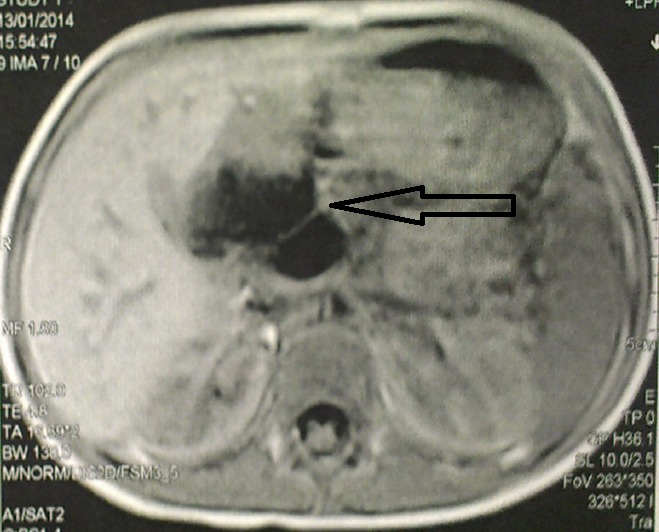
Abdominal MRI: cystic mass of the hepatic hilum in intimate contact with the duodenum, pulsing gallbladder and head of the pancreas

**Figure 4:**
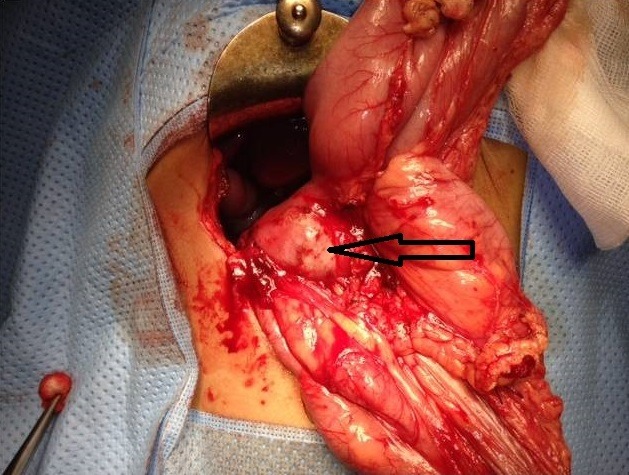
Whitish mass with cystic appearance of the head of the pancreas

**Figure 5:**
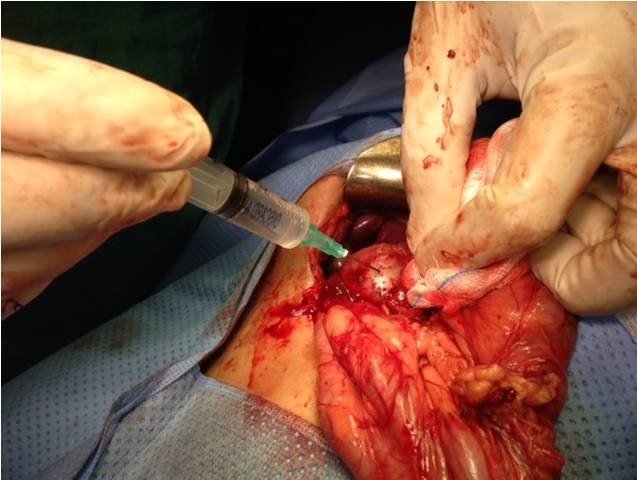
Puncture-emptying of the mass brings back clear liquid (rock water)

**Figure 6:**
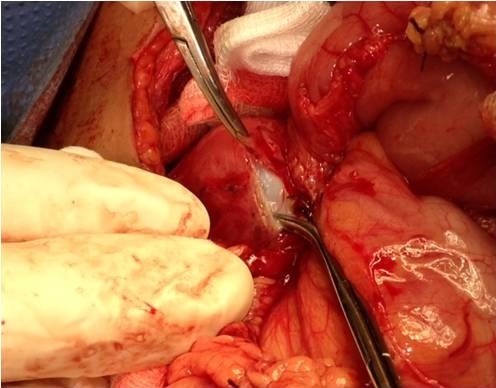
Resection of the protruding dome

**Figure 7:**
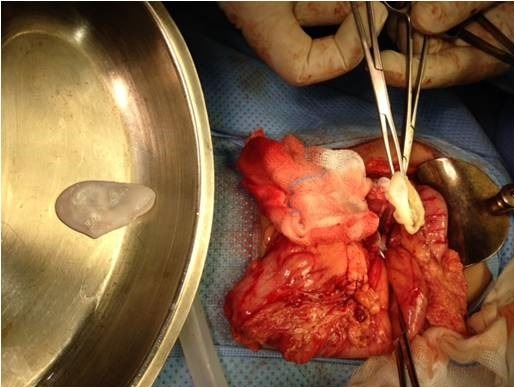
Extraction of three proliferating membranes

This is a 5-year-old girl from a rural area in the province of Kenitra (West Morocco) with no previous history hospitalized in April 2014 for epigastric pain during the past two weeks associated with dietary vomiting and diarrhea for a month and a half. The clinical examination was unusual. Abdominal ultrasound showed a multilocular hepatic cystic mass projecting at the level of the heterogeneous hypo echoic liver of the duodenum and gallbladder 46mm long major axis and coming into contact with the anterior surface of the head of the pancreas (Figure 1), in addition to a hydatid cyst, the diagnosis of a cyst of the bile duct or duodenal duplication. The abdominal angioscan supplemented by abdominal MRI confirmed the existence of this fluid mass of the left hypochondrium, showing a lesional cystic compartmentalized process in contact with the second duodenum, with a thickened wall septum making 57mm/31mm pushing back the gallbladder and the head of the pancreas of 10cm diameter, within which there existed a small round hypo echoic formation, evoking the vesicles girls. This formation appeared to depend on the head of the pancreas (Figure 2, Figure 3). The remainder of the radiological assessment did not detect other hydatid localization, in particular hepatic or pulmonary. Biology was normal except for lipase levels at 180 U/L (3 times normal). Hydatid serology (ELISA) was positive. The patient was operated on the right umbilical transverse route. Exploration found a cystic mass at the expense of the pancreatic lodge pushing the duodenum backwards without invading it (Figure 4). We carried out a coloepiploic detachment revealing the pancreatic seat of the mass. After protection of the surgical field and the abdominal wall by fields soaked in a scolicidal solution, draining of the mass was carried out, bringing clear liquid (rock water) (Figure 5) and a sterilization of the cyst, followed Resection of the protruding dome with extraction of 3 proliferating membranes and sterilization of the cyst with hypertonic serum (Figure 6, Figure 7). Then, we carried out an external drainage of the cystic cavity through a drain of Redon. Finally, the exploration of the peritoneal cavity did not objectify other localization. The operative sequences were simple; we did not notice a pancreatic fistula. We have prescribed Albendazole to our patient post-operatively for 6 months to further contribute to the sterilization of the cystic cavity and to prevent recurrence. Our patient was reviewed regularly at the consultation. With a follow-up of 2 years, ultrasound monitoring did not detect hydatid recurrence and immunology became negative.

## Discussion

The pancreas is an organ rarely infected with hydatid cysts and figures from the literature show an incidence ranging from 0.19% to 2% of the various sites of the hydatid disease [2, 3]. Inflammation of the pancreas occurs mainly by hematogenous or peri-pancreatic lymphatic invasion, but very rarely by retro-peritoneal propagation, as well as local spread through the pancreatic or biliary ducts has been suggested [4–9]. This location is isolated in 91% of cases with a slight predilection for the cephalic portion (57%) [2, 4]. The pancreatic hydatid cyst (KHP) was only exceptionally reported in children [4–9]. Preoperative diagnosis may be difficult due to the similarity of clinical presentation and radiological findings with other cystic lesions more commonly encountered in the pancreas [10]. This is the case of our observation where the radiological assessment evoked in addition to hydatid cysts of the pancreas the diagnoses of digestive duplication or cystic lymphangioma. The clinical presentation varies according to the size and anatomical seat of the cyst in the pancreas and the degree of biliopancreatic involvement [8]. Chronic epigastric pain, retentional jaundice (cephalic localization) or epigastric mass are the main clinical symptoms [11]. But most often, the diagnosis is made after complications of the cyst: suppuration [4], Fistulization in the main bile duct [4], portal hypertension by compression of the splenic vein [12], acute pancreatitis [13, 14].

Ultrasound, computed tomography (CT) and magnetic resonance imaging (MRI) make it easy to retain the diagnosis of a pancreatic cystic lesion [9, 11, 12]. Biologically, hydatid serology or ELISA for isolation of echinococcal antigens is positive in more than 85% of infected patients but is not always required during preoperative diagnosis. The diagnosis of certainty of the hydatid disease of the pancreas is carried out mainly during the surgery. The treatment of the hydatid cyst is essentially surgical. However, administration of Albendazole in pre- and post-operative for at least one month may help sterilize the cyst, reduce the risk of anaphylaxis and reduce the rate of postoperative recurrence [2, 11, 12]. Depending on the location of the cyst and the presence or absence of biliopancreatic fistula, various surgical techniques may be used [4, 11]. In fact, it is now accepted by the majority of authors that the morbidity of the drainage after resection of the protruding dome (pancreatic fistula) should be preferred for cornea-caudal localization, as for left-sided splenopancreatectomy [15]. On the other hand, for cephalic cysts, the reference treatment is a resection of the protruding dome associated in case of ductal fistula, with a kystodigestive anastomosis [13]. This derivation of the kystogastric or kystoduodenal anastomosis, or kystojejunal on the Y-handle, should be preferred to the external drainage or to the single resection of the protruding dome with or without epiploplasty because of the resulting morbidity [11, 12]. A hydatid cyst in the tail of the pancreas can be successfully treated with distal pancreatectomy [2]. Although the incidence of hydatid disease in the West and the rest of the world is low, we believe that immigration from endemic areas and the movement of people may increase the incidence of hydatid disease doctor the world might encounter in the future.

## Conclusion

Pancreatic hydatidosis is exceptional [1, 3]. It often poses diagnostic difficulties in the face of an isolated cystic lesion, despite the contribution and contribution of modern imaging techniques. The final diagnosis is intraoperative. The treatment of this condition is surgical [2]. The reference technique remains the resection of the protruding dome.

## Competing interests

The authors declare no conflict of interest.
